# Developing catalyst films of health experiences: an analysis of a robust multi-stakeholder involvement journey

**DOI:** 10.1186/s40900-022-00369-3

**Published:** 2022-07-29

**Authors:** Sarah Davis, Nancy Pandhi, Barbara Warren, Njeri Grevious, Madison Crowder, Haley Ingersoll, Elizabeth Perry, Andrew Sussman, Rachel Grob

**Affiliations:** 1 Center for Patient Partnerships, University of Wisconsin-Madison, 432 Lake St. Ste. 104, Madison, WI 53706 USA; 2 Primary Care Academics Transforming Healthcare (PATH), UW-Madison, Madison, USA; 3 Department of Family and Community Medicine, University of New Mexico School of Medicine, Albuquerque, USA; 4 Health Experiences Research Network (“HERN”), Madison, USA; 5 LGBT Programs and Policies in Mount Sinai’s Office for Diversity and Inclusion, Mount Sinai Health System, New York City, USA; 6 HERN National Patient Advisory Council, Madison, USA; 7 Xavier University, Cincinnati, USA; 8 Department of Family and Community Medicine, University of Wisconsin-Madison, Madison, USA

**Keywords:** Patient and public involvement, Co-design, Catalyst film, Trigger film, DIPEx methodology, Quality improvement, Visual participatory methods, Film, Video

## Abstract

**Background:**

Those whose lives are most directly impacted by health care—patients, caregivers, and frontline staff—are ideally situated to improve patient health care services and care quality. Despite a proliferation of literature on both Patient and Public Involvement (PPI) and clinical quality improvement (QI), concrete strategies regarding *how* to involve patients remain elusive.

**Aim:**

Research suggests catalyst films, comprised of rigorously-analyzed interview data from diverse patients about their experiences with health and health care (“catalyst films”) are a promising way to bring actionable patient feedback to QI. To date, such films have been crafted primarily by researchers. This project aimed to inform the science of engagement through analyzing how deliberate PPI informed the process of creating catalyst films.

**Methods:**

PPI methods included: research team norming activities through a project charter and role delineation process; key informant interviews; participant-ambassador videotaped interviews; clinician and research focus groups; and inclusion of advisors on the research team. Content studied for the analysis presented here included team meeting notes, interview and focus group transcripts, and documentation from a facilitated discussion about team processes. These data were analyzed to determine the impact of our PPI process. Member checking verified themes and lessons learned.

**Results:**

PPI shaped team deliberations and final products in substantial ways, including: what material to include in catalyst films and the tone they should convey; multiple issues regarding representation; and our collective understanding of how catalyst films could be used in the United States. Specific discussions addressed: how to include the optimal mix of interview segments that describe experiences with those that more directly point towards care improvement strategies; and how to balance positive and negative feedback from patients about experiences with care. Team process issues included ensuring equity in involvement despite team members having differing and sometimes multiple roles that complicated power dynamics and processes.

**Conclusions:**

Multiple forms and degrees of PPI resulted in significant influence on catalyst films and companion materials. Our project thus provides proof of concept for PPI in creation of video products for QI which have traditionally been crafted by researchers. The model we developed, and document in this paper, can be adapted by others creating research-derived video products. Our findings can also inform future research on how co-designing catalyst films enhances their value for QI and the application of co-designed catalyst film use in QI. Lastly, it can guide those engaged in QI and medical education in their selection of film products focused on patient experiences.

**Supplementary Information:**

The online version contains supplementary material available at 10.1186/s40900-022-00369-3.

## Abstract (summary)

Involving patients in care improvement efforts is valuable for improving the quality and safety of health care services because patients offer unique insights and are directly impacted by the system. Involving patients in these efforts can also inform better patient and family experiences. Studies have shown that using video interviews highlighting good and bad patient experiences in healthcare is one of the promising way to include a wider range of patient narratives and feedback in care improvement. Videos used in these situations are now called catalyst films, formerly known as trigger films. This paper describes how catalyst films are similar to and distinct from other film products used in research and improvement projects. It examines a process for equitably engaging a team of many different stakeholders—patients, providers, and researchers—to select video excerpts from existing research-based patient experience interviews to create catalyst films. It describes methods used to ensure robust input from all team members, so that all perspectives influence the catalyst films. The study concluded that patient and public involvement had significant impact on both the research process and the final products created. Our findings can equip those making or selecting films for use in improving health and social care to ensure films are patient informed. The paper concludes by offering limitations and recommendations for future research to advance the fields of patient and public involvement and quality improvement.

**Supplementary Information:**

The online version contains supplementary material available at 10.1186/s40900-022-00369-3.

## Background

Those whose lives are most directly impacted by health care—patients, caregivers, and frontline staff—are ideally situated to improve patient health care services and care quality. Indeed, multiple ongoing efforts exist to ensure that patients’ priorities are at the center of work on care quality. These include national calls from governments and professional associations [1–3], broad strategies such as the UK’s 4Pi National Involvement Standards [4], and successful efforts to include narratives in standardized survey instruments [5]. Despite these efforts, and the corresponding proliferation of publications and guidance documents about patient and public involvement (PPI), however, concrete strategies regarding *how* to involve patients in quality improvement (QI) remain elusive, with no one size fits all approach [6, 7].

There is concern that PPI requirements have led to a tick box mentality which front-loads involvement at the beginning of projects, thereby limiting development of best practices in later stages of research [8]. As a recent systematic review concluded, uncertainty about how to do this complex work well, and challenges regarding both how to include diverse patients, and how to form authentic partnerships, remain [9]. The field would benefit from more evidence about promising PPI strategies with respect to involving diverse stakeholders in all stages of research; creating and maintaining authentic relationships among stakeholders [8]; and addressing complex team power dynamics [10–12].

Collaboratively created videos, referred to as participatory videos, are a key component of participatory action research [4, 13] and a promising method of PPI in QI. While participatory videos are primarily used in community and international development [14], they also have been used in healthcare settings [15], for health promotion [16], and for clinical interventions [17]. Participatory visual methods also have proven useful for connecting with seldom heard voices [18] and have been shown to open dialogue about difficult topics [19], increase understanding among viewers about options for taking action [20], and facilitate discussions about how to move forward [21]. However, to our knowledge, few video products used in healthcare QI are co-created or edited by patients [22, 23].

Our overall aim in this paper is to document how different types and degrees of PPI can enhance the quality of a participatory video tool, termed a “catalyst film”, for quality improvement. To the best of our knowledge, the process of engaging stakeholders to create catalyst films has not been previously examined. We describe the process of involving multiple stakeholders—including patients, consumers, researchers and clinicians—to create catalyst films along with an accompanying guidebook that guides the film’s use in clinical quality improvement. We document contributions, decisions, and lessons learned from the expansive PPI processes we undertook as a team. Our analysis demonstrates the value that different types and degrees of PPI had on the creation of the film and its companion material.

We anticipate that our findings will be helpful to multiple audiences including: patients and consumers teaching and learning about engagement; researchers and others engaged in creation of research-derived video products designed to improve health and social care; and more broadly those wanting to translate their findings into quality improvement interventions. We also anticipate it will provide new insights for those selecting videos depicting patients’ experiences for QI and medical education; policymakers interested in methods for acquiring diverse and rigorously-analyzed accounts of lived experience (as opposed to arbitrary anecdotes); and all audiences committed to substantive PPI.

## Methods

### Conceptual models and influences

Our definition of PPI is the process of engaging multiple key stakeholders in efforts to improve health care delivery. This definition is consistent with that used by comparable studies that emphasize the importance of promoting a two-way relationship between patients and researchers or those engaged in Quality Improvement [24]. We were influenced in this work by co-design principles for quality improvement that emphasize “the interdependent work of users and professionals” to create health [25] and design for dissemination, an “active process that helps to ensure that …health interventions… are developed in ways that match well with adopters’ needs, assets, and time frames [26]”.

PPI methods used in this study included: research team norming activities through a project charter and role delineation process; key informant interviews; patient experience ambassador videotaped interviews; clinician and research focus groups; and inclusion of advisors on the research team.

### Catalyst films based on health experiences videos

Catalyst films have been created in the UK for more than a decade as part of Experience Based Co-Design (EBCD). EBCD is a method for improving services in partnership through a systematic, participatory process of reflection and collaboration [12]. Catalyst films have also been created from patient experience interviews gathered using the widely-adopted “Database of Individual Health Experiences” (DIPEx) methodology [27]. In the U.S., the DIPEx methodology is implemented by the Health Experiences Research Network or “HERN.” DIPEx interviews are used in a shortened version of EBCD called Accelerated EBCD (AEBCD) [28]. Analyzing interviews for themes relevant for QI and then assembling illustrative clips into “catalyst films” (also referred to as “trigger films” [29]) creates a unique tool which makes testimony about patients’ diverse experiences readily available for QI teams. Catalyst films created with DIPEx interviews from national studies can also offer perspectives that patients and families in local settings may be reluctant to share [28].

### Project team composition, inclusion of patients, and process

At the outset of the project, the Principal Investigators met to discuss what roles were needed on the project team that would best represent the different stakeholders intended as possible end users for the final catalyst film. Through this formal role delineation process, we determined that we would want to involve five groups: Patient and Consumer Advisors; Patient Experience Ambassadors, Clinicians, Researchers, and Research staff (Table 1). We recognized in constructing the team that several members might bring multiple perspectives from being part of multiple groups. For example, one group member was both a practicing clinician and a researcher with the original project. Another was a patient advisor and a previous patient experience ambassador. Patients were included at the two highest levels of engagement, “engage” and “partner [24]”.

**Table 1:** Stakeholder groups involved in the construction of the catalyst film

Stakeholders	Definition	Role
Patient and Consumer Advisors	Advisors with previous participation in Health Experiences Research Network (HERN) activities who bring subject matter, lived, and/or process experience to a project	As research partners, apply experience and training to center patient and family experiences and help the team “walk the talk” of co-design
Patient Experience Ambassadors	Research participants trained to disseminate health experiences research pertinent to their own health condition. They combine their own personalized lived experiences with knowledge of experiences of others who participated in the HERN study	Contributing at the “engage” level of PPI, shared insights on ideal content for catalyst films, and how catalyst films should be used. Contributed interviews, of which excerpts were used in final films
Clinicians	Clinicians offering their professional lens on current transformation in health care services regarding mental health, and the realities and constraints facing quality improvement efforts	Represent clinicians’ priorities and concerns and help the project team seek input from other clinicians (via focus groups) to develop catalyst films that will meet the needs of practicing clinicians
Principal investigators (PIs)	Project leaders with expertise in patient experiences research, quality improvement research, Patient and Public Involvement, and primary and secondary use of HERN materials	Provide oversight, ensure regulatory compliance, develop research protocols and lead research activities, set timelines and agenda for team discussions, ensure completion of final products, and link back to HERN
Additional Researchers	Researchers with subject matter expertise and unique access to clinicians at partner university	Review literature, contribute to protocol and liaise to, and co-lead, two clinician focus groups in a different geographical location
Research staff	Staff with variable abilities and assignments to ensure smooth project management and execution	Interview Patient Experience Ambassadors; conduct literature review; cut film clips and assemble drafts for project team review; manage project logistics, budget, and Institutional Review Board (IRB) process

A charter for the project was developed, clearly identifying its *co-design intention* through “deeply engaging key stakeholders through the entire life cycle of the project”. It also identified a “spirit of learning mindset to facilitate the capturing of lessons learned from our efforts” [Additional file 1: Appendix]. The stated outcomes were identified as: developing: 1) a catalyst film that could be used in accelerated EBCD quality improvement projects and 2) an accompanying user-friendly guidebook to maximize implementation of this method.

**Fig. 1:**
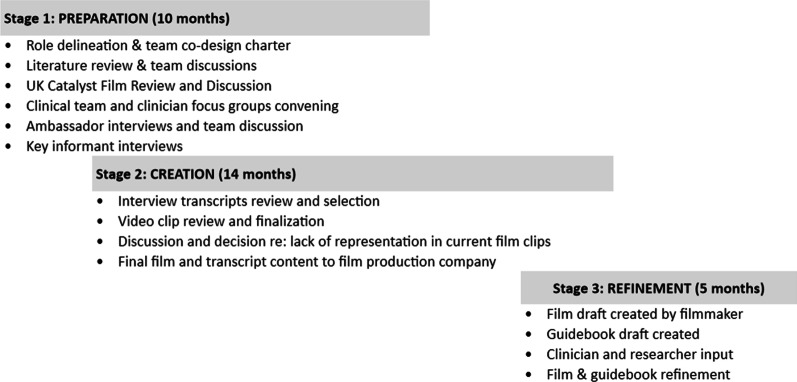
Timeline and research team activities, including patient and public involvement

The entire project team met frequently over a 19-month period. These meetings served as a forum for input and decisions that ultimately led to the creation of catalyst films and a guidebook. Team members spanned multiple geographical locations in the United States, so meetings were held using web-based technology platforms. Activities over the project period were divided into three stages: 1) Preparation: Developing a shared background for the team through literature review and viewing and discussing an existing UK catalyst film; holding key informant interviews; and conducting and analyzing individual interviews with patient experience ambassadors, and focus groups with clinicians and their teams; 2) Creation: Selecting material for catalyst films; and 3) Refinement: Finalizing the content of films and accompanying user guidebook. Our project involved patients at every step—from the very beginning through dissemination activities. Key activities are highlighted in Fig. 1. The project’s three stages overlapped and lasted 7 months longer than planned due to COVID-19.

### Key informant interviews to guide catalyst film development

We interviewed several key informants: an EBCD expert from the UK with ample experience making catalyst films; a researcher at the RAND corporation funded by the Robert Wood Johnson Foundation to implement an EBCD project in the US; and a group at Kaiser Permanente with expertise in patient engagement and EBCD. Several team members from each stakeholder group attended each meeting. One key takeaway from these conversations was that in the experience of informants, use of video in QI is almost always effective because first-hand testimony has a strong emotional impact. It is critical, however, that content balance positive and negative, leading with the former if possible so that the film is “modeling good care, so those watching will know if they are not living up to that example [30]”. Informants also highlighted that various film lengths can be effective, depending on context; that it is best practice to involve users throughout the entire design process; and that films can be most effective if they focus not just on experiences with services, but on what it is like to live with the particular illness or condition highlighted in the project. One informant also reminded us that “you are not disseminating research here,” rather, you are “trying to get people to do things differently on the basis of good research [30]”.

### Engaging additional stakeholders

To ensure robust input, we sought additional input from patient experience ambassadors, clinicians, and primary care teams not participating directly on our on-going project team. We performed individual interviews with five patient experience ambassadors. One patient ambassador echoed key informants’ visions of a catalyst film, saying: “It’s a call to action…it highlighted all the things that can be improved….and where it’s done right…” Another ambassador noted the value of reinforcing the range of patients’ experiences seeking care, stating “I think it’s a good way to remind… health professionals of the severity [and] novelty of this process [of seeking treatment] for some people.” Another noted that a strength of a catalyst film is the primacy of its interview content: “The key is authenticity…that [the film] is directly conveying what the participants have said, not trying to put it into any sort of narrative or message.”

We also conducted three focus groups with clinicians and clinical primary care teams. In these interviews and groups, we introduced the idea of catalyst films, viewed an example from the UK, and received feedback on the content and design of such films in the U.S. These viewers conveyed similar reflections as our ambassadors regarding the value of the film they viewed, particularly with respect to what they perceive such films add to QI processes. One noted that diversity of experiences is key: “Helpful to hear real stories and multiple stories– not pretend it’s all the same or ‘one size fits.’” Another commented on the film’s capacity to provide insight not otherwise available: “From a healthcare point of view, it’s nice to hear people’s opinions that you wouldn’t hear in person. It’s nice to be able to make improvements in care based on real experiences”.

### Analysis

Several sources of data were brought together to analyze the impact of our stakeholder engagement process. We performed a document analysis of team meeting notes to determine where non-researcher input had significantly shaped our final products. We reviewed our patient experience ambassador interview and focus group transcripts to determine what suggestions were made, and whether the final products reflected this feedback. Lastly, team members participated in a facilitated discussion in which they reflected individually and in groups on what went well as part of our team processes. Themes were extracted from this discussion and we engaged in reflective member checking, to both confirm validity and engage in continued learning as team members [31].

## Results

Our project resulted in seven separate catalyst films on Young Adults’ Experiences with Depression (length ranges from 3.5 to 19 min). Shorter films consist of a single subject focus on the following topics: Depression is Multifaceted, Stigma, Experiences of Patient-Centered Care and Coordination, Medication, Therapy and What Patients Want from Providers [32]. In addition to these films we created a comprehensive Guidebook to guide use of the film for QI that provides: context; an introduction to the use of catalyst films for various activities; guidance including worksheets, handouts, and sample agendas for film viewing and reflection; and links to additional resources on QI and PPI [33].

Stakeholder influence on the final products is documented in Table 2. As shown, PPI shaped team deliberations and the final products in substantial ways. An illustrative example was our extensive team conversations about representation of racial, ethnic, and gender diversity in the film. Early key informant and clinician input suggested the film needed to be “actionable”—to compel clinician viewers to act—in order to achieve a goal of catalyzing quality improvement. Specifically, we were advised to prioritize emotional salience over maximizing the number of included participants with diverse experiences, different from the intention of the original film clips from the web-based module on the HealthExperiencesUSA.org website. After viewing the first film draft, however, one of our advisor team members expressed concern over the lack of clear inclusion of Black, Indigenous, People of Color (BIPOC) individuals and representation of diverse gender identities. One of the challenges we faced was the fact that many original BIPOC participants decided not to be personally identifiable via video and instead provided audio or text interview material accompanied by a silhouette or other image. Our extensive deliberation resulted in the team re-reviewing video footage and associated transcripts to locate additional actionable material by BIPOC and those who identified as transgender or gender non-conforming. Our research associate who had conducted ambassador interviews for the project shared that she believed this new footage contained actionable material, beyond what was contained in the original interviews. This led us to ask an additional patient experience ambassador (who identifies as African American and also served as a patient advisor on this project) if they would be interested in being re-interviewed in an identifiable manner. They agreed and their footage coupled with newly found footage rounded out the representation in the films.

**Table 2:** Stakeholder influence on catalyst film construction

Project team questions	Stakeholder contributions	Decisions
Are there lessons from other teams creating catalyst films, or using EBCD, that can inform our own team process?	All project team members were invited to identify key informants to fully inform our process	Key informant input influenced our team to:
	Three informant groups were identified; one by a consumer advisor and two by the PIs	Prioritize emotional salience over strictly adhering to representation in order to maximize the ability to act on the information shared
	All team members were invited to attend key informant interviews; PIs, research staff and consumer advisors were represented at every meeting	Highlight positive as well as negative experiences
		Include footage discussing experiences with depression in general, not just with health care
How do we envision these films will be used in the United States?	All team members were invited to share insights and those with extensive experience in quality improvement (QI) in the United States shared observed barriers to patient participant involvement (PPI) generally	Clinician and PI experience drove the decision to “market” films for use broadly in QI and education in the US through description in a generalized guidebook, with a goal of maximizing uptake and spread
	Clinicians (in focus groups) and patient experience ambassadors (in interviews) were asked about their expectations for film usage	One consumer advisor was strongly opposed to this decision to not describe films as solely a product for Accelerated Experience Based Co-Design (AEBCD)
		Entire project team agreed with key informants who stressed that balance is important: “films can be most effective if they focus not just on experiences with services, but on what it is like to live with the particular illness or condition focused on in the project”
How should film design differ from UK films?	Team members, clinician focus groups and patient experience ambassadors viewed excerpts of a UK catalyst film and were asked about content and length for use in QI in the United States	Stakeholders agreed to two adaptations”
		US context requires shorter films
		Actors should not be used in films
Do we have sufficient actionable clips in existing and newly obtained footage?	PI/clinician and clinician re-coded transcripts for actionable material and then shared results with the whole team	Team included segments from re-coded original transcripts after extensive and iterative deliberation. We also noted specific limitations in existing footage, and made collective decision to include actionable footage from new interviews with patient experience ambassadors
	In a parallel process, research staff was re-interviewing patient experience ambassadors to inform use of film, and identified that interviews included additional actionable insights	
Which clips should make the final cut, and in what order should they be presented?	Multiple rounds of individual team member review and group discussions	Key informants stressed that it is critical that content balance positive and negative, leading with the former if possible so that the film is “modeling good care, so those watching will know if they are not living up to that example”
	Input from key informants about balancing emotional range of content	Patient experience ambassadors’ stressed that films should contain a message of “hope and change” to convey that young adults have expectations from their care teams and desire engagement
	Input from patient experience ambassadors about emphasizing “hope and change” in the films	
What should these films be called?	Identified that name “trigger film” used in the UK would not be appropriate for the US context, brainstormed other possible names, brought question to key informants	Decided on “catalyst film,” as these films are designed to rally viewers to action for improvement
How do we ensure ample representation while prioritizing the ability to act on the information shared?	Team reviewed multiple drafts of the film and identified missing experiences (e.g. LGBTQI and BIPOC representation)	Team agreed to review additional transcripts and clips to ensure representation without sacrificing the ability to act on the information shared
		PIs asked advisor/ambassador if she would consider being re-interviewed for additional on-camera BIPOC representation
		Advisor/ambassador agreed to be re-interviewed
How do we respond to the reality that many BIPOC participants elect to remain anonymous?	Team discussed options to expand non-anonymous clips and how to message about use of silhouettes	Advisor/ambassador agreed to have new footage used
	PI asked advisor/ambassador if they would be willing to be re-interviewed on camera	PIs agreed to find additional actionable clips of BIPOC participants
	Team discussed how best to visually represent participants who wished to remain anonymous	Team determined they did not want to use actors and discussed other options including mirroring choices made by participants on the HealthexperiencesUSA website (e.g. flowers, silhouettes) and agreed on the use of distinct and humanized silhouettes
		Team ultimately decided that statement about use of silhouettes would not elaborate on identity or reasons for decision to remain anonymous

Themes that emerged from our facilitated debrief session with team members suggested that our efforts to create a PPI-centered research process were largely successful. Team members cited effective organization and processes, mutual respect, and value for patient contributions and diverse representation as indicators of this intention. Advisors noted that they appreciated being able to efficiently focus on providing useful feedback, noting that it: “Worked amazingly well because the primary team picked up the majority of the work and [we] got to react to the material.” Individuals felt respected and believed in the project’s purpose. A clinician involved stated: “the space felt ‘safe and good’…. I felt free to say I don’t understand, and it was encouraged to do so.”

The entire project team valued the input gathered from patient experience ambassadors, clinicians, and staff, noting the extra content and perspective this added. There was global appreciation for team conversations about perspective taking and the importance of diverse representation in the catalyst films. Research associates specifically mentioned their appreciation for the Team Charter regarding PPI to “keep everyone on task.” Individually, team members universally indicated that the project was well organized with clear goals, roles, and tasks. The team generally worked well together: as stated by one team member, “People respected and tolerated tension…. [the PI] was able to be really direct while being generally considerate.”

Challenges were also noted. All team members had assigned roles, and yet many team members offered multiple perspectives, and all had to deal with geographic separation as we had two research sites and advisors located in additional regions. Our research team consisted of members with longer-term relationships, including consumer advocates with institutional power, and other multiple roles. This changed power dynamics that perhaps required different attention then when team member’s capital did not follow classic binary notions of researchers holding more power than advisors and ambassadors [11]. For example, our consumer advisor is a professional Lesbian, Gay, Bisexual, and Transgender (LGBT) advocate. She brought a strong voice for LGBT representation to the project, and our film coverage lacked sufficient footage to do justice to this community. The project PI, a member of the LGBT community herself, reflected that she felt stuck without a satisfying resolution as while we did find one additional piece of footage to include, it was not an ideal fit. She also believed she could have more effectively communicated in response to this legitimate critique from a powerful advisor.

Research staff also emphasized the realities of working with vast amounts of video footage and technical clip cutting procedures as major challenges. We had hoped to wrap this project up within 12 months, but faced both predictable and unexpected delays due to: the novel nature of the project; limitations of our underlying film footage; and the COVID pandemic, which temporarily changed team member priorities, including the filmmaker’s availability. Our purposeful inductive process—encouraging deep engagement—was not always easy for every member of the team to track as the project extended longer than anticipated. This approach resulted in gaps between meetings and some variable attendance.

## Discussion

Our project contributes to two lines of inquiry: the value of involving patients and consumers in the construction of video products to be used for QI, and the importance of acknowledging multiple roles individual research team members may simultaneously play. It contributes to conceptual development in PPI by documenting the process of engaging stakeholders while creating catalyst films. This documentation further provides proof of concept for PPI engagement in video product creation for use in healthcare QI which have traditionally been crafted without extensive, high-level, patient or public involvement. Our project also raises questions about how to best engage teams with people who have multiple identities and roles.

### Patients and consumers as film creators

This project enabled us to focus on the benefits and drawbacks of attempting to foster robust PPI in video product development. Film is a powerful medium to convey emotion and elicit empathy, and so it is well-suited for QI [34]. For authenticity and respectful conduct towards patients, we believe that such films should ideally be co-created by patients [23], in addition to featuring them—as is the case in participatory video [22] and catalyst films created by EBCD [12]. In our film creation process, we used PPI to ensure that our compilation of first person testimony conveyed insights of the whole team—patient and consumer advisors, patient experience ambassadors, clinicians, researchers, and research staff. The team collectively agreed that the selected clips represented key aspects of patients’ experiences relevant to QI. In this way, these patient narratives are more than ‘talking heads’ and may indeed introduce richer insights due to their multiple layers of curation [22], importantly by patients themselves.

Considering how to engage patients in more aspects of film creation seems appropriate and worthy of further study in light of both the power of patients’ narratives to inform QI, and the PPI field’s aspirations of co-design. Doing so may provide insight into the kind of expertise, methods, and ethics needed to elevate the role of patients in clinical quality improvement through the use of visual methods [22]. Participatory video has a long history of doing this in the community and international development domains [14]. Bringing this power to healthcare clinical quality improvement may prove to be one satisfying answer to *how* to involve patients for QI in a way that introduces new ways of thinking to catalyze transformation [22].

In many ways our video creation project mirrored EBCD processes where patients and consumers are involved at every step, thus enacting the ideal that “in this process they do not just say things, they do things as well; and they do them in person, not through some third party” [35]. We did fall short in some important ways, however. Since not all patient experience ambassadors and participants included in the film were members of the research team, some voices were mediated through representation by a designated team member. While the power of video [20] made it possible to literally bring these ambassadors’ voices into our discussions, these voices were only present at specific times and in hindsight, in limited ways; we should have included more video voices during our discussion and included patient experience ambassadors in the review of the final product.

### Multiple roles of team members

In all health research projects it is critical for team members to reflect on power dynamics in general, as well as the specific impact of imbalances between patients and researchers on PPI [11]. Issues of power are particularly salient for co-design projects involving vulnerable and disadvantaged populations [12], such as the one we report on here. Our project surfaced the need to broaden the study of team power dynamics to include an evaluation of the multiple roles team members may play as a critical contribution to the emerging evidence base around PPI. Future exploration of power relations in PPI would benefit from studying diverse research teams. Crosswalking learnings from team science—with related challenges navigating conflict and maintaining team cohesion across disciplinary differences—may be valuable for both areas of practice [36].

### Reflections

Our findings are subject to limitations. Our experience may be more applicable to projects building off DIPEx research, than to those collecting data de novo, given that we used existing film footage and therefore needed to work with the limitations of that footage. Teams with patients creating a video product from scratch may experience less constraints and challenges with representation. The fact that we had already formed a core research team and subteams, may also limit its generalizability. Newer teams may need to invest in establishing trust and will invariably encounter unique power dynamics. We underutilized our patient experience ambassadors and could have enhanced PPI further by including additional voices from the video footage in our deliberations. Lastly, we only received feedback on the guidebook that accompanies the film from the research team; we would widen and deepen PPI in the future on companion materials as well.

## Conclusions

Multiple forms of PPI resulted in significant influence on catalyst films and companion materials. The model we developed for PPI in the creation of video products can be adapted by others engaged in creation of research-derived video products designed to improve health and social care. It can also guide those engaged in QI and medical education in their selection of film products regarding patient experiences—to ensure that viewers are seeing through the eyes of actual patients and consumers. Future projects could also benefit from using patient experience video footage, itself, as a rich source of PPI in research discussions. Future research on benefits and drawbacks of multiple roles played by individual team members, and how this reality can enhance collective agency and maximize PPI influence on final products, would contribute to the rich research base on PPI. And future research on how co-designing catalyst films enhances their value for QI and the application of co-designed catalyst film use in QI would benefit the field of quality improvement.

## Supplementary Information

**Additional file 1.** Team Charter.

## Data Availability

Existing video datasets analysed during the current study are available on the HealthexperiencesUSA website at https://www.healthexperiencesusa.org/Depression-in-Young-Adults/overview. The datasets generated, and transcribed used during the current study are available from the corresponding author on reasonable request.

## Abbreviations

PPI: Patient and public involvement
QI: Quality improvement
DIPEx: Database of Individual Health Experiences
EBCD: Experience-based co-design
AEBCD: Accelerated Experience Based Co-Design
HERN: Health Experiences Research Network
BIPOC: Black, Indigenous, People of Color
