# Supplementary material for: Developing catalyst films of health experiences: an analysis of a robust multi-stakeholder involvement journey
Source: Res Involv Engagem. 2022 Jul 29;8:34. doi: 10.1186/s40900-022-00369-3 (PMC9335457; doi:10.1186/s40900-022-00369-3)
Supplement: Supplementary file 1 — Additional file 1. Team Charter. [file 40900_2022_369_MOESM1_ESM.docx]

**Health Experiences Experience-Based Co-Design Catalyst Film Project**

**DRAFT Team Charter**

***Catalyst films*** are designed to jump start a health care quality improvement (QI) process by infusing it with patient experiences from the very beginning, so that transformations are grounded in values of the people at the center of health care.

***Experience-based co-design (EBCD)* *of Health Care Services*** is an approach that enables staff and patients (or other service users) to co-design services and/or care pathways, together in partnership. It involves making catalyst films during the co-design process.^[[1]](#footnote-1)^

***Co-design*** is an approach to participatory design (traditionally of a new product) that seeks to actively involve all stakeholders in a process to help ensure the results meet their needs and is useable.^[[2]](#footnote-2)^

***Accelerated EBCD (AEBCD),*** modifies EBCD that includes making films with the use of existing film(s) from an archive, which have been found to trigger a similar response in patients despite requiring half the resources of the full approach.^1^ In this project, we are making catalyst films that will be used in AEBCD QI efforts, and creating materials to support their use and additional film creation in the United States.

**Whole Team - DRAFT**

We will follow co-design principles to create short (5-7 minute) “Catalyst Films” to be used for Quality Improvement (QI) projects. This means we will deeply engage key stakeholders through the entire project lifecycle, including patients who are well-versed in the Young Adults with Depression Module and the Health Experiences Research Network (HERN), and clinicians with experience to inform relevant QI efforts. Our engagement will take several forms: (1) Patient Partner and Clinician Partner representation on our research team; (2) Focus group(s) with/input from patients who participated in the Young Adults with Depression Module; (3) Focus group(s) with/input from clinicians invested in QI in the mental health space; and (4) Key informant interviews with researchers who have created catalyst films using CD methods. This CD process is intended to result in a product that can be used in an accelerated EBCD quality improvement process.

Throughout the project, we will endeavor to employ a “spirit of learning mindset” to facilitate the capturing of lessons learned from our efforts. We will used these lessons towards at least three goals; (1) to course correct as needed during the project, (2) to inform a guidebook for use of catalyst films for QI efforts, and (3) to facilitate future catalyst film development.

**Patient Partner Sub-Team - DRAFT**

Patient Partners understand the value of having patient members of research teams and the responsibility to “represent” to the extent possible, patients’ priorities and/or to help the team seek patient input. Patient Partners are in no way solely responsible for ensuring that health experiences are properly understood and represented in the project. Rather, they offer unique expertise and experience to make sure that the breadth and depth of patient and family experiences are considered and that the team is “walking the talk” of co-design. The patients engaged in this process – both as Patient Partners on the research team, and as participants in focus group(s) have all participated in the making of the Young Adults with Depression Module and/or HERN activities so bring both subject matter, lived experience, and process experience to this project.

**Clinician Sub-Team – DRAFT**

Clinician Partners offer the clinician lens on current transformation in health care services regarding mental health, and the realities and constraints facing quality improvement efforts. Clinician Partners attempt to “represent” clinicians’ priorities and concerns and help the research team seek input from other clinicians and develop catalyst films that will meet the needs of busy, practicing clinicians. They will help the team describe the AEBCD process in a user-friendly guidebook to maximize implementation of this method. Clinicians engaged at Clinician Partners have experience in mental health integration in primary care. Clinicians engaged in focus group(s) will have experience serving on QI teams, sharing their experiences and priorities so that the catalyst films, and accompanying materials, we produce are of maximum value to busy QI teams.

1. Point of Care Foundation, Experience-Based Co-Design Toolkit. <https://www.pointofcarefoundation.org.uk/resource/experience-based-co-design-ebcd-toolkit/step-by-step-guide/1-experience-based-co-design/> [↑](#footnote-ref-1)
2. Prestantia Health, Experience-Based Co-Design – A Toolkit for Australia. https://ahha.asn.au/EBCDtoolkit/files/downloads/EBCD%20toolkit%20Final.pdf [↑](#footnote-ref-2)
